# A Fast Image Alignment Approach for 2D Classification of Cryo-EM Images Using Spectral Clustering

**DOI:** 10.3390/cimb43030117

**Published:** 2021-10-18

**Authors:** Xiangwen Wang, Yonggang Lu, Jiaxuan Liu

**Affiliations:** 1 School of Information Science and Engineering, Lanzhou University, Lanzhou 730000, China; wangxw2018@lzu.edu.cn (X.W.); jxliu2013@lzu.edu.cn (J.L.); 2 College of Computer Science and Engineering, Northwest Normal University, Lanzhou 730070, China

**Keywords:** cryo-electron microscopy, single-particle reconstruction, class averaging, image alignment, 2D interpolation, spectral clustering

## Abstract

Three-dimensional (3D) reconstruction in single-particle cryo-electron microscopy (cryo-EM) is a significant technique for recovering the 3D structure of proteins or other biological macromolecules from their two-dimensional (2D) noisy projection images taken from unknown random directions. Class averaging in single-particle cryo-EM is an important procedure for producing high-quality initial 3D structures, where image alignment is a fundamental step. In this paper, an efficient image alignment algorithm using 2D interpolation in the frequency domain of images is proposed to improve the estimation accuracy of alignment parameters of rotation angles and translational shifts between the two projection images, which can obtain subpixel and subangle accuracy. The proposed algorithm firstly uses the Fourier transform of two projection images to calculate a discrete cross-correlation matrix and then performs the 2D interpolation around the maximum value in the cross-correlation matrix. The alignment parameters are directly determined according to the position of the maximum value in the cross-correlation matrix after interpolation. Furthermore, the proposed image alignment algorithm and a spectral clustering algorithm are used to compute class averages for single-particle 3D reconstruction. The proposed image alignment algorithm is firstly tested on a Lena image and two cryo-EM datasets. Results show that the proposed image alignment algorithm can estimate the alignment parameters accurately and efficiently. The proposed method is also used to reconstruct preliminary 3D structures from a simulated cryo-EM dataset and a real cryo-EM dataset and to compare them with RELION. Experimental results show that the proposed method can obtain more high-quality class averages than RELION and can obtain higher reconstruction resolution than RELION even without iteration.

## 1. Introduction

Cryo-electron microscopy (cryo-EM) has become a recognized powerful technique in structural biology for three-dimensional (3D) structure determination of biological macromolecules, supramolecular complexes, and subcellular structures [1,2,3]. It does not need crystallization and has been widely used to study large macromolecular complexes that are difficult to be crystallized. The goal of cryo-EM 3D reconstruction is to reconstruct a high-resolution estimation of the 3D structure of the molecule from a set of micrographs [4,5,6]. Cryo-EM can be used to investigate complete and fully functional macromolecular complexes in different functional states, providing a richness of biological insight [7,8]. Cryo-EM has made tremendous progress in the past few years [9,10]. Owing to these exciting new developments, cryo-EM was selected by Nature Methods as the “Method of the Year 2015”, and the Nobel Prize in Chemistry 2017 was awarded to Jacques Dubochet, Joachim Frank, and Richard Henderson “for developing cryo-electron microscopy for the high-resolution structure determination of biomolecules in solution” [5].

As one of the major cryo-EM techniques, single-particle reconstruction has become one of the most successful techniques for structural biology [11,12,13]. Single-particle reconstruction using cryo-EM has been undergoing fast transformations, leading to an abundance of new high-resolution structures and reaching close to atomic resolution [14,15]. In the single-particle reconstruction, the same macromolecule is projected from various unknown directions, and the final 3D structure of the macromolecule can be reconstructed from the two-dimensional (2D) projection images using the estimated projection directions in 3D space [16,17]. One of the major challenges to be overcome in the single-particle reconstruction of biological samples is to estimate the projection directions of the projection images [18,19]. However, due to the very low signal-to-noise ratio (SNR) of the projection images caused by low-dose electron radiation, it is usually difficult to obtain the correct estimation of the projection directions. Consequently, the single-particle 3D reconstruction of cryo-EM is a very challenging task [20,21].

Class averaging in single-particle cryo-EM is an important procedure for producing high-quality initial 3D structures and discarding invalid particles or contaminants [22]. It organizes a dataset by grouping together the particles corresponding to the same (or quite similar) projection directions. Each group of cryo-EM projection images is regarded as a class and is averaged to produce an averaged image called a class average. By averaging, the random noise in the background tends to be canceled, and the features of interest in the projection images are reinforced by each other as the number of superimposed projection images becomes large [23,24]. Class averages can be used to improve ab initio modeling in cryo-EM. They can also be applied for discovering heterogeneity or symmetricity as well as for separating particles into subgroups for additional analysis [25].

Different solutions have been proposed for solving the 2D class averaging problem in cryo-EM [26,27,28,29,30,31]. Some popular cryo-EM software packages, such as cryoSPARC [32] and RELION [33,34,35] have implemented 2D class averaging. RELION uses a maximum likelihood expectation maximization (ML-EM) 2D classification procedure to infer parameters for a statistical model from the data. The ML-EM scheme has suffered less from initial reference bias, but it is computationally expensive. The iterative stable alignment and clustering (ISAC) algorithm [36] is another famous 2D class averaging method. ISAC relies on a modified k-means clustering method and the concepts of stability and reproducibility, which can extract validated, homogeneous subsets of projection images. ISAC is also time consuming.

Image alignment is a fundamental step in the class averaging procedure [37,38]. The cryo-EM projection images are required to be identified and rotationally and translationally aligned to distinguish among different classes. After alignment, the cryo-EM projection images with nearly the same projection directions are grouped in the 2D classification step. Well-aligned cryo-EM projection images with correct in-plane rotations and translational shifts in the x-axis and y-axis directions can improve the accuracy of the 2D classification [39]. Correctly classifying the cryo-EM projection images into homogeneous groups renders the satisfactory determination of the preliminary 3D structures [40]. Although translational invariant and rotational invariant image representation methods have been used in cryo-EM, they usually are not powerful enough to discover subtle differences between projection images [41]. It is necessary to design efficient image alignment algorithms to find the best alignment parameters and generate high-quality class averages.

Image alignment is aimed at estimating three alignment parameters: a rotation angle and two translational shifts in the x-axis and y-axis directions. Image rotational alignment and translational alignment in real space need too many iterations to compute the alignment parameters, and the calculated alignment parameters are integers. In Fourier space, alignment parameters can be computed directly without enumeration. In this paper, an efficient image alignment algorithm using the 2D interpolation in the frequency domain of images is proposed to improve the estimation accuracy of alignment parameters, which can obtain subpixel and subangle accuracy. Specifically: (1) for image rotational alignment, two images are transformed by polar fast Fourier transform (PFFT) to calculate a discrete cross-correlation matrix, and then the 2D interpolation is performed around the maximum value in the cross-correlation matrix. The rotation angle between the two images is directly determined according to the position of the maximum value in the cross-correlation matrix after interpolation. (2) For image translational alignment, all operation steps are consistent with image rotational alignment, where fast Fourier transform (FFT) is used instead of PFFT. (3) For image alignment with rotation and translation, only a few iterations of combined rotational and translational alignment are needed to align images. Furthermore, the proposed algorithm and a spectral clustering algorithm [42] are used to compute class averages for single-particle 3D reconstruction. The main contributions of this paper are summarized as follows:

- 2D interpolation in the frequency domain is used to improve the estimation accuracy of the alignment parameters, which can obtain subpixel and subangle accuracy.
- The alignment parameters of rotation angles and translational shifts in the x-axis and y-axis directions can be computed directly in Fourier space without enumeration, which is very fast.
- A spectral clustering algorithm is used for the unsupervised 2D classification of single-particle cryo-EM projection images.

The rest of this paper is organized as follows: In Section 2, the proposed image alignment algorithm is described in detail, including the image rotational alignment, the image translational alignment, and image alignment with rotation and translation. The unsupervised 2D classification of cryo-EM projection images performed by using a spectral clustering algorithm is also introduced. In Section 3, the flexibility and performance of the proposed image alignment algorithm are demonstrated through three datasets, including a Lena image, a simulated dataset of cryo-EM projection images, and a real dataset of cryo-EM projection images. The single-particle 3D reconstruction using produced class averages is also performed and compared with RELION. Finally, this paper is concluded in Section 4.

## 2. Materials and Methods

**Figure 1:**
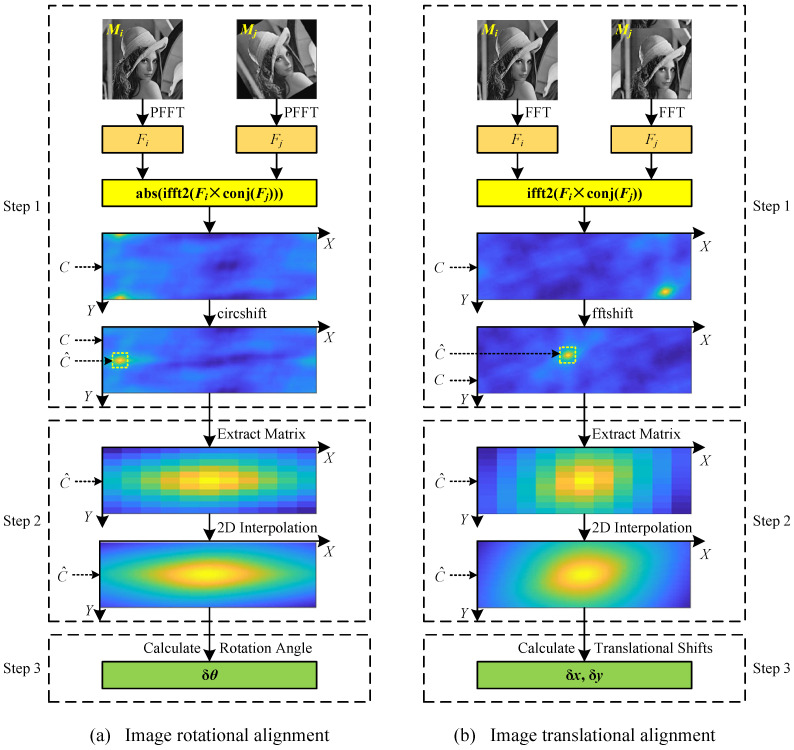
The diagrams of the proposed image rotational and translational alignment algorithms using 2D interpolation in the frequency domain of images. (**a**) Image rotational alignment. (**b**) Image translational alignment.

In this section, the proposed image alignment algorithm is demonstrated in detail, including (1) image rotational alignment; (2) image translational alignment; and (3) image alignment with rotation and translation. The diagrams of the proposed image rotational and translational alignment algorithms using 2D interpolation in the frequency domain of images are shown in Figure 1. Then the proposed algorithm and a spectral clustering algorithm are used to compute class averages.

### 2.1. Image Rotational Alignment

Image rotational alignment is one of the basic operations in image processing. The rotation angle between two images can be estimated either in real space or in Fourier space. In real space, image rotational alignment is a rotation-matching process, that is, an exhaustive search. An image is rotated in a certain step size, and the similarity between the rotated image and the reference image is calculated. When the image is rotated for one circle, the index corresponding to the maximum similarity is the final estimated rotation angle between the two images. This method is simple, but it is time consuming and inaccurate. Assuming the search step size is *p*, image rotational alignment in real space requires 360/p rotation-matching calculations. Although the coarse-to-fine search method can be used, it still needs to be calculated many times.

In this paper, the image rotational alignment is implemented in Fourier space without rotation-matching iteration, which is a direct calculation method. In general, the cryo-EM projection images are square; therefore, only the rotational alignment of the square image is considered. For two images $M_{i}$ and $M_{j}$ of size m×m, the proposed image rotational alignment method is illustrated in Figure 1a. In the rest of this paper, the proposed image rotational alignment algorithm is represented as function rotAlign(·,·). There are three key steps in the image rotational alignment algorithm:

- Step 1: Calculate a cross-correlation matrix using PFFT. Firstly, images $M_{i}$ and $M_{j}$ are transformed by PFFT to obtain two corresponding spectrum maps $F_{i}$ and $F_{j}$ with the size of ⌊m/2⌋×360. Then, the cross-correlation matrix *C* is calculated according to:
  (1) $$C=abs(ifft2\left(F_{i}\times conj\left(F_{j}\right)\right))$$
  where abs(·) is an absolute value function, ifft2(·) is a 2D inverse fast Fourier transform function, and conj(·) is a complex conjugate function. These functions have been implemented in MATLAB. The values in matrix *C* need to be circularly shifted by ⌊m/4⌋ positions to exchange rows to horizontally center the large values in matrix *C*, where the function circshift implemented in MATLAB can be used. The size of the cross-correlation matrix *C* is ⌊m/2⌋×360.
- Step 2: 2D interpolation around the maximum value in the cross-correlation matrix *C*. The rotation angle δθ of the image $M_{j}$ relative to the image $M_{i}$ can be roughly determined according to the position of the maximum value in the cross-correlation matrix *C* on the x-axis. The rotation angle calculated by this method is an integer. In order to calculate the rotation angle more accurately, the 2D interpolation is performed around the maximum value in the cross-correlation matrix *C*. Specifically, an 11 * 11 matrix $\hat{C}$ centered on the maximum value in the matrix *C* is extracted from the matrix *C* (see the dotted box in Figure 1a), and then the 2D interpolation is performed in the matrix $\hat{C}$. Theoretically, any interpolation method can be used in the proposed algorithm. In this paper, the spline interpolation is used to perform the 2D interpolation, which has been implemented in MATLAB as function interp2 with parameter ‘spline’. After 2D interpolation, the size of the matrix $\hat{C}$ becomes 101×101.
- Step 3: Calculate the rotation angle. The rotation angle δθ can be directly calculated according to the position of the maximum value in the matrix $\hat{C}$ after interpolation on the x-axis. Generally, the rotation angle δθ of an image is in the range of $\left[-180^{\circ },180^{\circ }\right]$, so δθ needs to be corrected according to:
  (2) $$\delta \theta =\left\{\begin{array}{cc} \delta \theta , & if\;0^{\circ }\leq \delta \theta \leq 180^{\circ }\\ \delta \theta -360^{\circ }, & if\;180^{\circ }<\delta \theta <360^{\circ } \end{array}\right.$$

### 2.2. Image Translational Alignment

Image translational alignment can also be realized in real space or Fourier space. In real space, image translational alignment is also an exhaustive search, and it is more complex than image rotational alignment. For two images $M_{i}$ and $M_{j}$ of size m×m, it needs to compute the similarity between each row (column) of $M_{i}$ and each row (column) of $M_{j}$ and then determines the translational shift δx in the x-axis direction and the translational shift δy in the y-axis direction according to the maximum similarity. Therefore, the image translational alignment in real space requires 2×m×m similarity calculations. In addition, the translational shifts estimated in real space are integers, which are not accurate enough.

Similar to image rotational alignment, in this paper, the image translational alignment is implemented in Fourier space. It is a direct calculation method without enumeration. For two images $M_{i}$ and $M_{j}$ of size m×m, the proposed image translational alignment method is illustrated in Figure 1b. In the rest of this paper, the proposed image translational alignment algorithm is represented as function shiftAlign(·,·). There are three key steps in the image translational alignment algorithm:

- Step 1: Calculate a cross-correlation matrix using FFT. Firstly, images $M_{i}$ and $M_{j}$ are transformed by FFT to obtain two corresponding spectrum maps $F_{i}$ and $F_{j}$ with size of m×m. Then, the cross-correlation matrix *C* is calculated according to:
  (3) $$C=ifft2(F_{i}\times conj\left(F_{j}\right))$$
  The values in matrix *C* need to be shifted to center the large values in matrix *C*, where the function fftshift implemented in MATLAB can be used. The size of the cross-correlation matrix *C* is m×m.
- Step 2: Two-dimensional interpolation around the maximum value in the cross-correlation matrix *C*. The translational shifts δx and δy of the image $M_{j}$ relative to the image $M_{i}$ in the x-axis and y-axis directions can be roughly determined according to the position (x,y) of the maximum value in the cross-correlation matrix *C* on the x-axis and y-axis, respectively. The translational shifts calculated by this method are integers. In order to calculate the translational shifts more accurately, just as with the image rotational alignment described in Section 2.1, the 2D interpolation is performed around the maximum value in the cross-correlation matrix *C*, where the spline interpolation is used. Specifically, an 11×11 matrix $\hat{C}$ centered on the maximum value in the matrix *C* is extracted from the matrix *C* (see the dotted box in Figure 1b), and then the 2D interpolation is performed in the matrix $\hat{C}$. After 2D interpolation, the size of the matrix $\hat{C}$ becomes 101×101.
- Step 3: Calculate the translational shifts. The translational shifts δx and δy of the image $M_{j}$ relative to the image $M_{i}$ are directly calculated according to the position (x,y) of the maximum value in the matrix $\hat{C}$ after interpolation on the x-axis and y-axis, respectively:
  (4) $$\left\{\begin{array}{c} \delta x=\lfloor m/2\rfloor -x+1\\ \delta y=\lfloor m/2\rfloor -y+1 \end{array}\right.$$

### 2.3. Image Alignment with Rotation and Translation

Image alignment with rotation and translation is a fundamental but challenging step in class averaging. It is the coupling of image rotational alignment and image translational alignment and generally requires iterations. In this paper, an efficient image alignment algorithm using the 2D interpolation in the frequency domain of images is proposed, which is listed in Algorithm 1. The functions rotAlign(·,·) and shiftAlign(·,·) represent the image rotational alignment algorithm described in Section 2.1 and the image translational alignment algorithm described in Section 2.2, respectively. The functions imrotate(·,·) and imshift(·,·) represent the image rotation operation and image translation operation, respectively. For each iteration, the test image *M* is first rotationally aligned and then translationally aligned to calculate the three alignment parameters. The final alignment parameters Δθ, Δx, Δy, and the original test image *M* are used to calculate the final aligned image $M^{A}$, reducing the error accumulation caused by interpolation calculation in image rotation and translation. When the alignment parameters δθ, δx, and δy during the iteration remain unchanged, the iteration can be terminated ahead of time. According to our experience, the program converges within ten iterations in the majority of cases. In addition, there is no complicated operation in the algorithm. It can efficiently and effectively align two images. Therefore, the proposed algorithm can be used to align a large number of cryo-EM projection images.
**Algorithm 1:** Image alignment algorithm using 2D interpolation in the frequency domain.
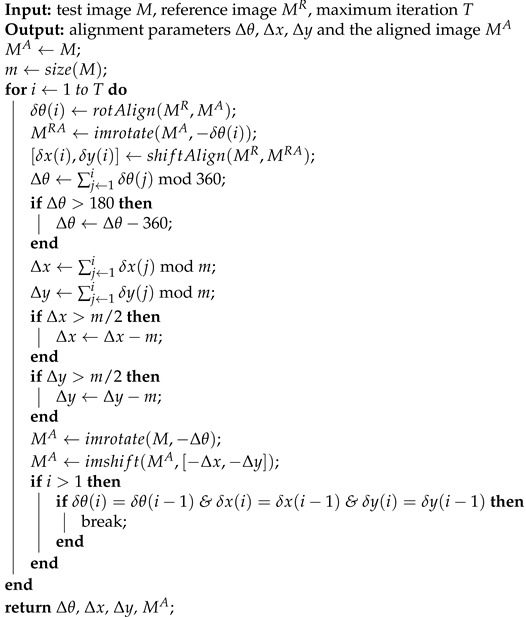


### 2.4. Class Averaging

**Figure 2:**
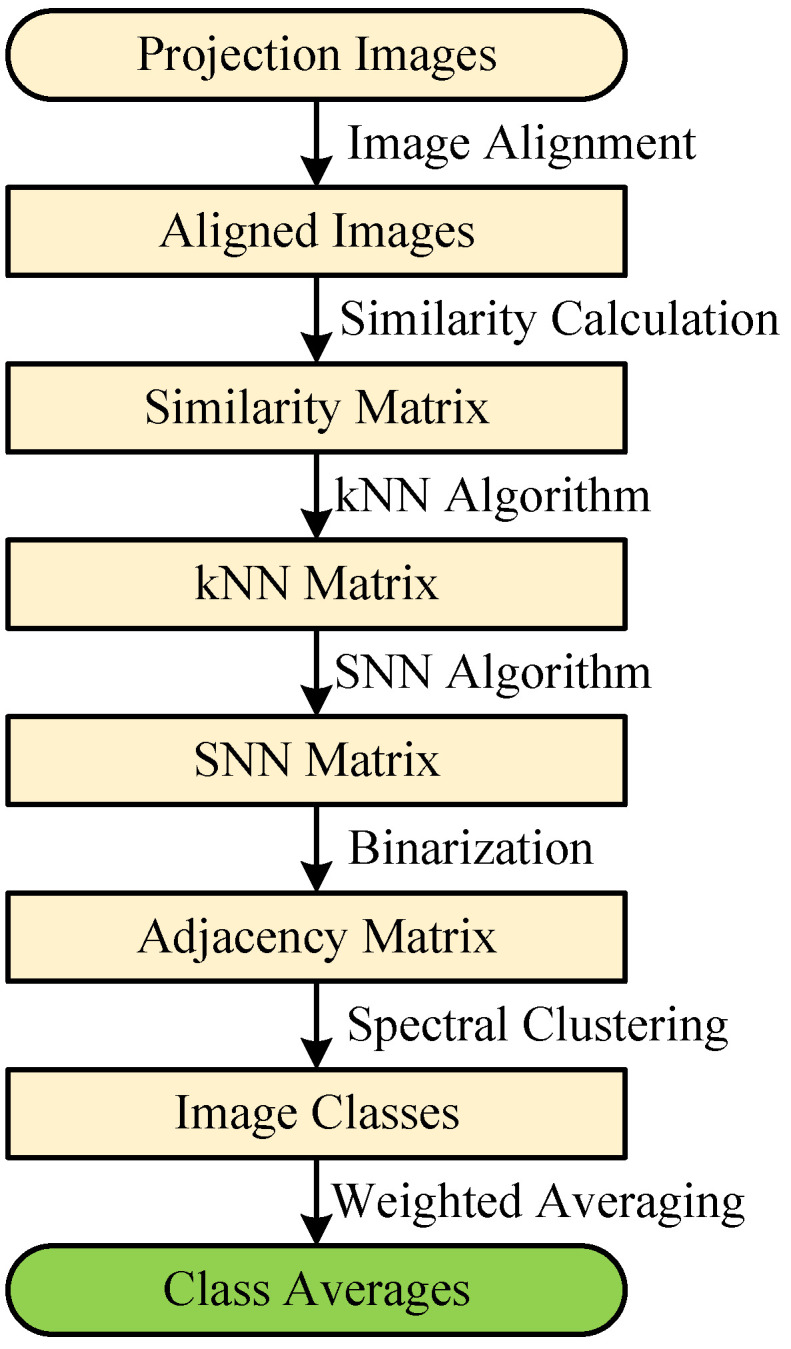
A diagram of the calculation process of the class averaging.

In this paper, the proposed image alignment algorithm and a spectral clustering algorithm [42] are used to implement class averaging. The calculation process of the class averaging is shown in Figure 2. First of all, all the cryo-EM projection images are aligned by the proposed image alignment algorithm to calculate the similarity matrix *S* between them. As in many studies [5,25], the 2D correlation coefficient is used to compute the similarity:

(5) $$S\left(i,j\right)=\frac{\sum_{x=1}^{m}\sum_{y=1}^{m}\left(M_{i}\left(x,y\right)-\bar{M_{i}}\right)\left(M_{j}\left(x,y\right)-\bar{M_{j}}\right)}{\sqrt{\left(\sum_{x=1}^{m}\sum_{y=1}^{m}{\left(M_{i}\left(x,y\right)-\bar{M_{i}}\right)}^{2}\right)\left(\sum_{x}^{m}\sum_{y}^{m}{\left(M_{j}\left(x,y\right)-\bar{M_{j}}\right)}^{2}\right)}}$$

where $M_{i}\left(x,y\right)$ and $M_{j}\left(x,y\right)$ are the pixel values of images $M_{i}$ and $M_{j}$, respectively. $\bar{M_{i}}$ and $\bar{M_{j}}$ are the mean values of the pixel values of images $M_{i}$ and $M_{j}$, respectively. *m* is the size of the projection image in one dimension.

Afterward, the similarity matrix *S* is converted into an adjacency matrix AM using a k-nearest neighbor (kNN) algorithm [43] and a shared nearest neighbor (SNN) algorithm [44]. Specifically, the matrix SNN, which is used to represent the number of shared near neighbors between projection images $M_{i}$ and $M_{j}$, is calculated as follows:

(6) $$SNN\left(i,j\right)=\left|KNN(i)\cap KNN(j)\right|$$

where KNN(i) and KNN(j) are the sets of *k* nearest neighbors of projection images $M_{i}$ and $M_{j}$, respectively, which can be found according to the similarity matrix *S*. The matrix SNN is converted into an adjacency matrix AM by binarization:

(7) $$AM\left(i,j\right)=\left\{\begin{array}{cc} 1, & SNN\left(i,j\right)>N_{S}\\ 0, & \mathrm{otherwise} \end{array}\right.$$

where $N_{S}=5$ is the threshold parameter used to represent at least $N_{S}$ shared nearest neighbors between projection images $M_{i}$ and $M_{j}$. The empirical value of parameter *k* in the kNN algorithm can be calculated adaptively according to the total number of projection images *N*:

(8) $$k=\sqrt{N}+N_{S}$$

Finally, the adjacency matrix AM is used as the input of the normalized spectral clustering algorithm [45] to perform unsupervised classification. Projection images grouped in a class are aligned and weighted averaged to produce a class average. Assuming that the *j*th class contains $N_{j}$ projection images, the class average $M_{j}^{AVG}$ can be calculated as:

(9) $$M_{j}^{AVG}=\frac{1}{\sum_{i=1}^{N_{j}}S\left(i,j\right)}\sum_{i=1}^{N_{j}}\left(M_{i}S\left(i,j\right)+M_{j}\right)$$

where S(i,j) is the similarity between the projection image $M_{j}$ that is closest to the cluster center of the *j*th class and the projection image $M_{i}$ that is aligned with $M_{j}$ in the *j*th class.

## 3. Results and Discussion

In this section, some experiments are performed to demonstrate the performance of the proposed image alignment algorithm. Firstly, the proposed image alignment algorithm is used to estimate alignment parameters. Secondly, the proposed image alignment algorithm and the normalized spectral clustering algorithm with adjacency matrix are used to produce class averages for reconstructing the preliminary 3D structure. The performance of the image alignment algorithm in Fourier space with and without 2D interpolation is compared. The running time of image alignment in Fourier space and real space is also compared. The reconstruction results are compared with RELION [35]. For the convenience of description, in the rest of this paper, the image alignment algorithm in Fourier space using the 2D interpolation is named IAFI; the image alignment algorithm in Fourier space without interpolation is named IAF; and the image alignment algorithm in real space is named IAR. The search step in IAR is 1.

### 3.1. Feasibility of the Image Alignment Algorithm

**Figure 3:**
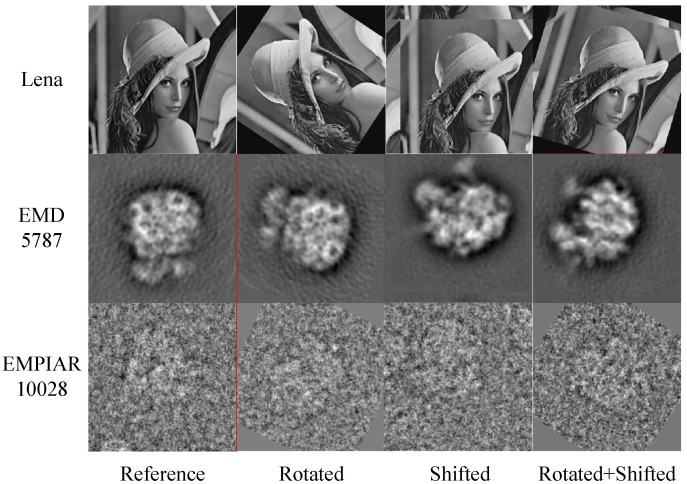
Samples of the test image.

The proposed image alignment algorithm was performed on three datasets to estimate alignment parameters of rotation angles and translational shifts in the x-axis and y-axis directions. The first dataset contains a Lena image of size 256×256 pixels. The second dataset contains 100 clean simulated cryo-EM projection images of size 128×128 pixels projected from the published cryo-EM structure EMD5787 [46] with random projection directions. The third dataset contains 100 real cryo-EM projection images selected randomly from the picked particles of EMPIAR10028 [47], which were down sampled to 180×180 pixels. Three simulations were designed to test the performance of the proposed image alignment algorithm: (1) test images were only rotated; (2) test images were only shifted; and (3) test images were firstly shifted and then rotated. Figure 3 shows some test images used in the simulations. All simulations in this subsection were run on MATLAB R2018b on a six-core system with 16 GB RAM in a Windows 10 environment.

The first simulation estimates the rotation angles between the reference images and the test images. For the first dataset, the Lena image is rotated 100 times randomly in the range of $\left[-180^{\circ },180^{\circ }\right]$ to generate 100 test images. For other datasets, each projection image is rotated randomly in the range of $\left[-180^{\circ },180^{\circ }\right]$ to generate a test image. The ground-truth rotation angles were set to only one decimal place. The rotation angles between images were estimated using the image rotational alignment algorithm described in Section 2.1. Table 1 shows the frequency distribution of the absolute error in degrees between the estimated and the ground-truth rotation angles for different datasets. It can be seen that both the IAFI algorithm and the IAF algorithm can estimate the rotation angles with small errors. The errors of the IAFI algorithm are less than $0.5^{\circ }$ for all datasets while the errors of the IAF algorithm are greater than $0.5^{\circ }$ but less than $1^{\circ }$ in a few cases. The total error of the IAFI algorithm is smaller than that of the IAF algorithm for all datasets. It indicates that the proposed image rotational alignment algorithm can estimate the rotation angles between images with high accuracy.

Table 2 shows the running time in seconds for different image rotational alignment algorithms to run 100 times. It can be seen that image rotational alignment in Fourier space is much faster than that in real space. In addition, for all of these three algorithms, the larger the image size, the more time they take to rotationally align images. The 2D interpolation calculation in IAFI is very fast, and the estimated rotation angles using IAFI are more accurate than using IAF. This shows that the proposed image rotational alignment algorithm is very efficient.

The second simulation estimates the translational shifts in the x-axis and y-axis directions between the reference image and the test image. For the first dataset, the Lena image was shifted 100 times randomly in the range of [−m/10,m/10] in the x-axis and y-axis directions to generate 100 test images. For other datasets, each projection image was shifted randomly in the range of [−m/10,m/10] to generate a test image. The ground-truth translational shifts were set to only one decimal place. The translational shifts between images were estimated using the image translational alignment algorithm described in Section 2.2. Table 3 and Table 4 show the frequency distribution of the absolute error in pixels between the estimated and the ground-truth translational shifts in the x-axis and y-axis directions, respectively, for different test images. It can be seen that the absolute errors for both the IAFI algorithm and the IAF algorithm are within 1 pixel. In particular, the IAFI algorithm can estimate the translational shifts almost exactly for all of these three datasets. It indicates that the proposed image translational alignment algorithm can accurately estimate translational shifts between images.

Table 5 shows the running time in seconds for different image translational alignment algorithms to run 100 times. It can be seen that image translational alignment in Fourier space is much faster than that in real space. In addition, for all of these three algorithms, the larger the image size, the more time they take to translationally align images. This shows that the proposed image translational alignment algorithm is very efficient.

Image alignment with both rotation and translation is more difficult than only rotation or translation. The third simulation estimates the alignment parameters including rotation angles and translational shifts in the x-axis and y-axis directions between the reference image and the test image. In the single-particle 3D reconstruction, most particles were almost centered in the particle picking procedure, which means only a small number of translational shifts are required. So, a small number of translational shifts were set on the test images in this simulation. For the first dataset, the Lena image was firstly shifted 100 times randomly in the range of [−m/20,m/20] in the x-axis and y-axis directions and then rotated randomly in the range of $\left[-180^{\circ },180^{\circ }\right]$ to generate 100 test images. For other datasets, each projection image was firstly shifted randomly in the range of [−m/20,m/20] in the x-axis and y-axis directions and then rotated randomly in the range of $\left[-180^{\circ },180^{\circ }\right]$ to generate a test image. The ground-truth rotation angle and translational shifts were set to only one decimal place. The maximum iteration was set as 10.

Table 6 shows the frequency distribution of the absolute error in degrees between the estimated and the ground-truth rotation angles for different test images. It can be seen that both the IAFI algorithm and the IAF algorithm can estimate the rotation angle with small errors, and the total error of the IAFI algorithm is smaller than that of the IAF algorithm for all test images. The frequency distribution of the absolute error in pixels between the estimated and the ground-truth translational shifts in the x-axis and y-axis directions for different test images are shown in Table 7 and Table 8, respectively. It can be seen that the IAFI algorithm can estimate the translational shifts with smaller errors than the IAF algorithm. It should be noted that for the EMPIAR10028 dataset, in rare cases, the estimated rotation angle is wrong (the error greater than $5^{\circ }$), resulting in the estimated translational shifts also being wrong (the error greater than 5 pixels). This indicates that the proposed image alignment algorithm is very effective for estimating alignment parameters between images.

Table 9 shows the distribution of the number of the final iterations. It can be seen that both the IAFI algorithm and the IAF algorithm converge within 10 iterations for all test images in most cases. Generally, the IAFI algorithm and the IAF algorithm require five iterations. On the whole, the proposed image alignment algorithm can accurately align images within 10 iterations.

### 3.2. Single-Particle 3D Reconstruction

The proposed image alignment algorithm and the normalized spectral clustering algorithm [45] with adjacency matrix were used to produce class averages, which were later used for reconstructing the preliminary 3D structure. The simulated single-particle cryo-EM projection images of EMD5787 [46] and the real cryo-EM projection images of EMPIAR10028 [47] were used in this experiment. The projection images are aligned using the proposed image alignment algorithm. The reconstruction results using the image alignment algorithms IAFI and IAF were compared with RELION [35], which was embedded in the SCIPION software framework [48,49]. This experiment was performed using the ASPIRE software package (http://spr.math.princeton.edu/, accessed on 18 August 2021). The preliminary 3D structure was reconstructed from the generated class averages using the common-lines-based angular reconstruction method [50], which was implemented as the function “cryo_estimate_mean” in the ASPIRE software package. The projection direction of cryo-EM projection images was estimated using the synchronization algorithm [51], where the common lines between class averages were estimated using our proposed weighted voting algorithm [52]. All cryo-EM 3D structures were visualized by the UCSF ChimeraX software [53,54].

**Figure 4:**
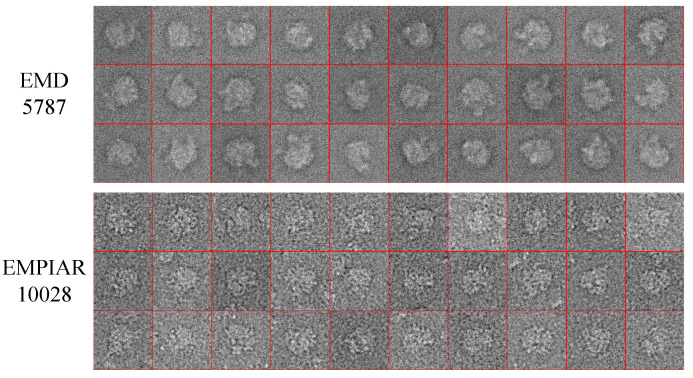
Samples of projection images in the cryo-EM datasets of EMD5787 and EMPIAR10028.

Firstly, 10,000 clean centered EMD5787 projection images with the size of 128×128 pixels were generated through random rotation matrices corresponding to random projection directions that were uniformly distributed over the rotation group SO(3). The clean centered projection images are shifted randomly in the range of [−m/20,m/20] in the x-axis and y-axis directions. The additive Gaussian white noise with the fixed SNR=0.1 was added to the clean shifted projection images to generate the final noisy projection images. The SNR is defined as follows:

(10) $$SNR=\frac{\mathrm{var}(signal)}{\mathrm{var}(noise)}$$

where var is the variance (energy), signal is the clean projection image, and noise is the noise realization of that projection image. Meanwhile, 10,000 real cryo-EM projection images were selected randomly from the picked particles of EMPIAR10028 and were down-sampled to 180×180 pixels. The projection images in EMPIAR10028 were globally phase flipped so that the molecule corresponds brighter pixels and the background corresponded to darker pixels. Figure 4 shows some projection images in the cryo-EM datasets of EMD5787 and EMPIAR10028.

**Figure 5:**
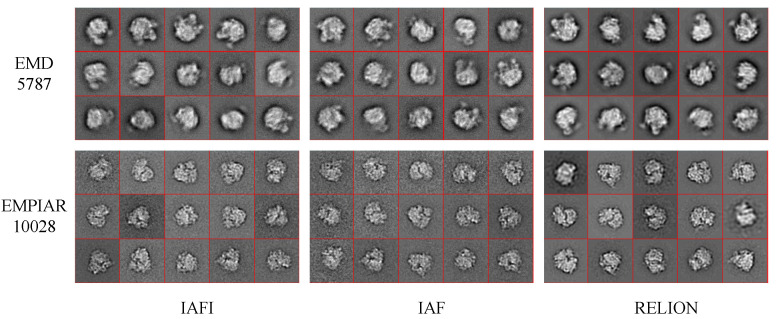
Samples of the class averages were produced by different methods for the cryo-EM datasets of EMD5787 and EMPIAR10028.

Then, the cryo-EM projection images were aligned using the image alignment algorithms IAFI and IAF. The similarity matrix between the aligned projection images was converted into an adjacency matrix using the kNN and SNN algorithms, which was input into the normalized spectral clustering algorithm for 2D classification. The 10,000 aligned projection images were classified into 100 classes. The projections classified into the same class were aligned and weighted averaged to produce a class average. Figure 5 shows some class averages produced by different methods for the cryo-EM datasets of EMD5787 and EMPIAR10028. Not all the 100 class averages for each dataset were usable for 3D reconstruction, and some bad class averages needed to be excluded. Table 10 shows the number of good class averages that were manually selected for 3D reconstruction.

**Figure 6:**
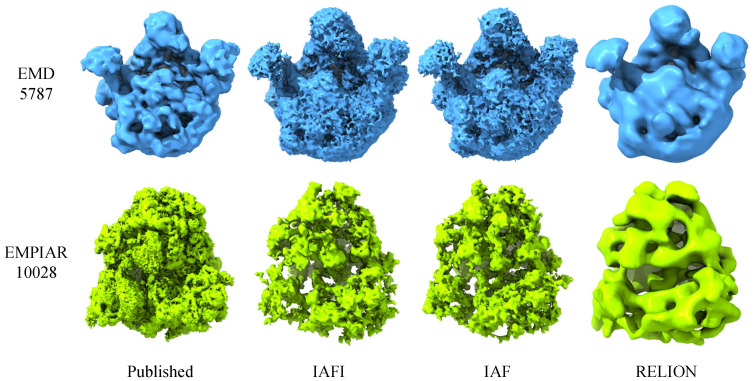
The published cryo-EM structures (EMD5787 [46] and EMD2660 [47]) and the reconstructed preliminary 3D structures using different methods for the cryo-EM datasets of EMD5787 and EMPIAR10028.

Finally, the preliminary 3D structure was reconstructed from the selected good class averages. Figure 6 shows the published cryo-EM structures (EMD5787 [46] and EMD2660 [47]) and the reconstructed preliminary 3D structures using different methods for the cryo-EM datasets of EMD5787 and EMPIAR10028. The voxel dimensions of 3D structures for these two datasets are 2.54×2.54×2.54 Å and 2.68×2.68×2.68 Å, respectively. These reconstructed 3D structures were masked with the radius m×0.4 and aligned with the corresponding published structures. The reconstruction results of RELION were achieved after 25 iterations, while the reconstruction results of IAFI and IAF were directly computed without iteration. It can be seen that the reconstructed 3D structures are similar to the corresponding published structures. This indicates that the calculated class averages were effective in the single-particle 3D reconstruction of cryo-EM, and have certain advantages in comparison with RELION.

**Figure 7:**
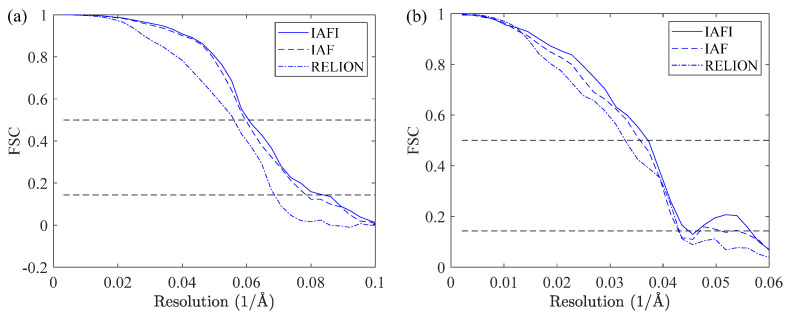
FSC curves of the preliminary 3D structures were reconstructed from the cryo-EM datasets of EMD5787 (**a**) and EMPIAR10028 (**b**) using different methods.

Figure 7 shows the corresponding Fourier shell correlation (FSC) curves [55] of the reconstructed preliminary 3D structures from different datasets. Figure 7a shows the FSC curves of the reconstructed EMD5787 structures, which were computed against the published cryo-EM structure of EMD5787 [46] shown in Figure 6. Figure 7b shows the FSC curves of the reconstructed 3D structures from EMPIAR10028, which were computed against the published cryo-EM structure of EMD2660 [47] shown in Figure 6. It can be seen that using the IAFI algorithm can obtain a higher reconstruction resolution than using the IAF algorithm. This is because the IAFI algorithm can estimate the alignment parameters between projection images more accurately than the IFA algorithm, so that the projection images can be aligned and classified more accurately and finally produce higher quality class averages for 3D reconstruction. In addition, the reconstruction accuracy of the proposed 2D classification method using the image alignment algorithms IAFI and IAF is higher than that of RELION. This is because the proposed 2D classification method can produce more high-quality class averages than RELION. This further indicates that the proposed 2D classification method is practicable in the single-particle 3D reconstruction of cryo-EM and can achieve satisfactory results. In summary, the proposed 2D classification method is effective for the single-particle 3D reconstruction of cryo-EM, and the well-aligned cryo-EM projection images can improve the accuracy of the 2D classification and further improve the accuracy of 3D reconstruction.

## 4. Conclusions

The class averaging technique is useful for the 2D analysis of electron micrographs as well as in single-particle cryo-EM 3D reconstruction. Image alignment is a fundamental step in the class averaging procedure. In this paper, an efficient image alignment algorithm using the 2D interpolation in the frequency domain is proposed. The proposed image alignment algorithm was tested on a Lena image and two datasets of cryo-EM projection images for estimating alignment parameters. The simulation results show that the image alignment algorithm in Fourier space using the 2D interpolation can achieve higher estimation accuracy for alignment parameters than the image alignment algorithm in Fourier space without interpolation. It is also found that image alignment in Fourier space is faster than that in real space. In addition, the proposed image alignment algorithm and the normalized spectral clustering algorithm were used to produce class averages for reconstructing preliminary 3D structures. Results on datasets of simulated and real cryo-EM projection images indicate that the proposed method can be used to improve the resolution of the reconstructed preliminary structure. The main drawback of this study is that the contrast transfer function (CTF) parameters were not considered during the class averaging, which may limit the potential benefits of using the proposed method. In future work, we will integrate the CTF parameters into the class averaging procedure and apply the proposed method to large-scale datasets of cryo-EM projection images for high-resolution cryo-EM 3D reconstruction.

## Figures and Tables

**Table 1:** The frequency distribution of the absolute error in degrees between the estimated and the ground-truth rotation angles for different test images that were only rotated.

Error	Lena		EMD5787		EMPIAR10028	
	IAFI	IAF	IAFI	IAF	IAFI	IAF
[0, 0.5)	100	91	100	84	100	94
[0.5, 1]	0	9	0	16	0	6
total error	6.0	24.2	11.3	27.8	4.4	23.0

**Table 2:** The average running time in seconds for different image rotational alignment algorithms to run 100 times for different test images that were only rotated.

Datasets	Image Size	IAFI	IAF	IAR
Lena	256×256	0.6161	0.5435	377.4849
EMD5787	128×128	0.3941	0.3172	89.0824
EMPIAR10028	180×180	0.5218	0.4318	159.9434

**Table 3:** The frequency distribution of the absolute error in pixels between the estimated and the ground-truth translational shifts in the x-axis direction for different test images that were only shifted.

Error	Lena		EMD5787		EMPIAR10028	
	IAFI	IAF	IAFI	IAF	IAFI	IAF
[0, 0.5)	100	87	100	86	100	87
[0.5, 1]	0	13	0	14	0	13
total error	0.5	28.0	0.0	23.8	4.2	24.8

**Table 4:** The frequency distribution of the absolute error in pixels between the estimated and the ground-truth translational shifts in the y-axis direction for different test images that were only shifted.

Error	Lena		EMD5787		EMPIAR10028	
	IAFI	IAF	IAFI	IAF	IAFI	IAF
[0, 0.5)	100	94	100	91	100	89
[0.5, 1]	0	6	0	9	0	11
total error	0.5	25.2	0.0	26.0	3.9	26.2

**Table 5:** The running time in seconds for different image translational alignment algorithms to run 100 times for different test images that were only shifted.

Datasets	Image Size	IAFI	IAF	IAR
Lena	256×256	0.8403	0.7820	1102.3793
EMD5787	128×128	0.2545	0.2057	193.7869
EMPIAR10028	180×180	0.3979	0.3579	726.7303

**Table 6:** The frequency distribution of the absolute error in degrees between the estimated and the ground-truth rotation angles for different test images that were firstly shifted and then rotated.

Error	Lena		EMD5787		EMPIAR10028	
	IAFI	IAF	IAFI	IAF	IAFI	IAF
[0, 0.5)	100	87	99	89	86	73
[0.5, 1]	0	13	1	11	3	14
(1, 5]	0	0	0	0	0	0
≥5	0	0	0	0	11	13
total error	12.4	23.7	6.0	25.1	831.7	1031.3

**Table 7:** The frequency distribution of the absolute error in pixels between the estimated and the ground-truth translational shifts in the x-axis direction for different test images that were firstly shifted and then rotated.

Error	Lena		EMD5787		EMPIAR10028	
	IAFI	IAF	IAFI	IAF	IAFI	IAF
[0, 0.5)	100	86	100	93	88	77
[0.5, 1]	0	14	0	7	1	10
(1, 5]	0	0	0	0	2	2
≥5	0	0	0	0	9	11
total error	1.6	27.0	0.0	24.0	304.4	449.5

**Table 8:** The frequency distribution of the absolute error in pixels between the estimated and the ground-truth translational shifts in the y-axis direction for different test images that were firstly shifted and then rotated.

Error	Lena		EMD5787		EMPIAR10028	
	IAFI	IAF	IAFI	IAF	IAFI	IAF
[0, 0.5)	100	84	100	91	88	81
[0.5, 1]	0	16	0	9	1	5
(1, 5]	0	0	0	0	0	1
≥5	0	0	0	0	11	13
total error	2.9	26.8	0.0	24.8	285.9	533.3

**Table 9:** The distribution of the number of final iterations.

Iteration	Lena		EMD5787		EMPIAR10028	
	IAFI	IAF	IAFI	IAF	IAFI	IAF
3	4	8	11	10	6	14
4	6	36	10	46	12	37
5	57	51	59	33	31	26
6	26	4	12	10	28	11
7	7	0	8	1	10	2
8	0	1	0	0	2	0
9	0	0	0	0	1	1
10	0	0	0	0	10	9
mean iteration	5.26	4.55	4.96	4.46	5.84	4.99

**Table 10:** The number of good class averages for 3D reconstruction.

Datasets	IAFI	IAF	RELION
EMD5787	100	100	47
EMPIAR10028	88	83	25

## Data Availability

Publicly available datasets were used in this study. The published cryo-EM density maps EMD5787 and EMD2660 can be download from http://www.emdataresource.org/. The dataset EMPIAR10028 can be download from https://www.ebi.ac.uk/empiar/.
